# Two-year outcomes of ultrasound-guided percutaneous tenotomy for long head of the biceps tendinopathy

**DOI:** 10.1007/s00264-026-06751-0

**Published:** 2026-02-16

**Authors:** Süleyman Kaan Öner, Enes Alptekin Canli, Mehmet Korkmaz, Nihat Demirhan Demirkıran

**Affiliations:** 1 Department of Orthopaedics and Traumatology, Kütahya Sağlık Bilimleri Üniversitesi, Kütahya, Turkey; 2 Department of Radiology, Kütahya Sağlık Bilimleri Üniversitesi, Kütahya, Turkey

**Keywords:** Long head of the biceps tendon, Ultrasound-guided tenotomy, Percutaneous tenotomy, Biceps tendinopathy, Shoulder pain

## Abstract

**Background:**

The long head of the biceps tendon (LHBT) is a common source of anterior shoulder pain, particularly in older adults, and may persist despite conservative treatment. Arthroscopic tenotomy is effective but requires an operating room, anaesthesia, and postoperative restrictions, which may be suboptimal in elderly or comorbid patients. Ultrasound-guided percutaneous LHBT tenotomy has emerged as a minimally invasive alternative, yet long-term clinical outcomes remain insufficiently reported. This study aimed to evaluate two-year pain, functional, and sleep-quality outcomes following ultrasound-guided percutaneous LHBT tenotomy in patients with isolated LHBT tendinopathy.

**Methods:**

This retrospective case series included 51 consecutive patients (mean age 61.8 ± 4.8 years) with MRI-confirmed isolated LHBT tendinopathy who underwent ultrasound-guided percutaneous tenotomy between 2022 and 2024. Pain (VAS), functional scores (ASES and Constant–Murley), and sleep quality (PSQI) were assessed at baseline and at three, six, 12, and 24 months. Repeated-measures ANOVA or Friedman tests were used for longitudinal analysis, with effect sizes reported as partial eta-squared. Complications and patient satisfaction were recorded at the final follow-up.

**Results:**

All outcome measures improved significantly at each postoperative time point compared with baseline (p < 0.001). Mean VAS decreased from 6.84 ± 1.29 to 2.16 ± 0.89 at 24 months (η^2^ = 0.71), with 92.1% achieving the minimal clinically important difference (MCID). Functional outcomes improved markedly (ASES: 35.7 → 85.1; Constant–Murley: 60.4 → 82.5), both with large effect sizes (η^2^ = 0.68 and 0.64). PSQI improved from 9.2 ± 3.1 to 4.8 ± 2.2 (η^2^ = 0.56), reducing clinically significant sleep disturbance from 78.4% to 29.4%. Four patients (7.8%) developed asymptomatic Popeye deformity; no major complications occurred. Patient satisfaction at 24 months was 88.2%.

**Conclusions:**

Ultrasound-guided percutaneous LHBT tenotomy is a safe, minimally invasive, and effective procedure that provides durable improvements in pain, function, and sleep quality over two years, with a low complication rate. It represents a valuable alternative to arthroscopic tenotomy in appropriately selected patients.

## Introduction

The long head of the biceps tendon (LHBT) is a frequent source of anterior shoulder pain and commonly accompanies rotator cuff pathology [1, 2]. Its anatomical course through the bicipital groove makes it vulnerable to tendinopathy and associated symptoms [3]. Although most patients initially respond to conservative treatment, a considerable proportion continue to experience persistent pain requiring interventional management [4].

Tenotomy and tenodesis are established surgical options; however, arthroscopic techniques necessitate an operating room setting, anaesthesia, and postoperative recovery that may be suboptimal for elderly or comorbid patients [5–7]. In recent years, ultrasound-guided percutaneous LHBT tenotomy has emerged as a minimally invasive alternative that allows real-time visualization and can be performed under local anaesthesia [8, 9]. Despite promising feasibility data, mid- and long-term clinical outcomes remain insufficiently reported.

The purpose of this study was to evaluate two-year pain, functional, and sleep-quality outcomes following ultrasound-guided percutaneous tenotomy for LHBT tendinopathy. We hypothesized that the procedure would yield durable improvements with a low complication profile.

## Materials and Methods

### Study Design

This study was conducted as a retrospective case series **(**Level IV evidence) evaluating clinical, functional, and sleep-related outcomes following ultrasound-guided percutaneous long head of the biceps tendon (LHBT) tenotomy. Although all procedures were performed using a standardized institutional protocol and follow-up assessments were collected at predetermined intervals, the data review and analysis were performed retrospectively. Therefore, the study represents a retrospective observational analysis of consecutively treated patients. Ethical approval was obtained from the institutional review board of the hosting centre, and all patients provided written informed consent prior to inclusion.

## Patient Selection

Patients undergoing ultrasound guided percutaneous LHBT tenotomy between January 2022 and December 2024 were screened.

### Inclusion Criteria

• Age 40–75 years.

• Clinical diagnosis of LHBT tendinopathy (positive Speed and/or Yergason tests).

• Ultrasound-confirmed LHBT pathology (tendinosis, sheath effusion, or partial tear).

• MRI demonstrating isolated LHBT tendinopathy with no rotator cuff tear.

• Failure of ≥ three months of conservative therapy.

• Availability of complete clinical data for ≥ 24 months.

### Exclusion Criteria

• Full-thickness rotator cuff tears requiring repair.

• Prior surgery on the affected shoulder.

• Glenohumeral instability, infection, or tumor.

• Neuromuscular disorders affecting shoulder function.

• Known allergy to local anaesthetics.

### MRI Diagnostic Criteria

MRI evaluation followed a standardized shoulder protocol. Isolated LHBT tendinopathy was diagnosed when the following objective criteria were present:

- Increased intra-tendinous T2/PD-FS signal within the intra-articular LHBT.
- Tendon thickening ≥ 6 mm at the bicipital groove level.
- Longitudinal split/delamination changes on axial or sagittal sequences.
- Sheath effusion > 2 mm circumferentially.
- Absence of rotator cuff tears, confirmed on two orthogonal sequences.

All MRIs were assessed by a single fellowship-trained musculoskeletal radiologist who was blinded to clinical data to minimize interpretation bias.

### Pre-Procedural Ultrasonography

**Fig. 1:**
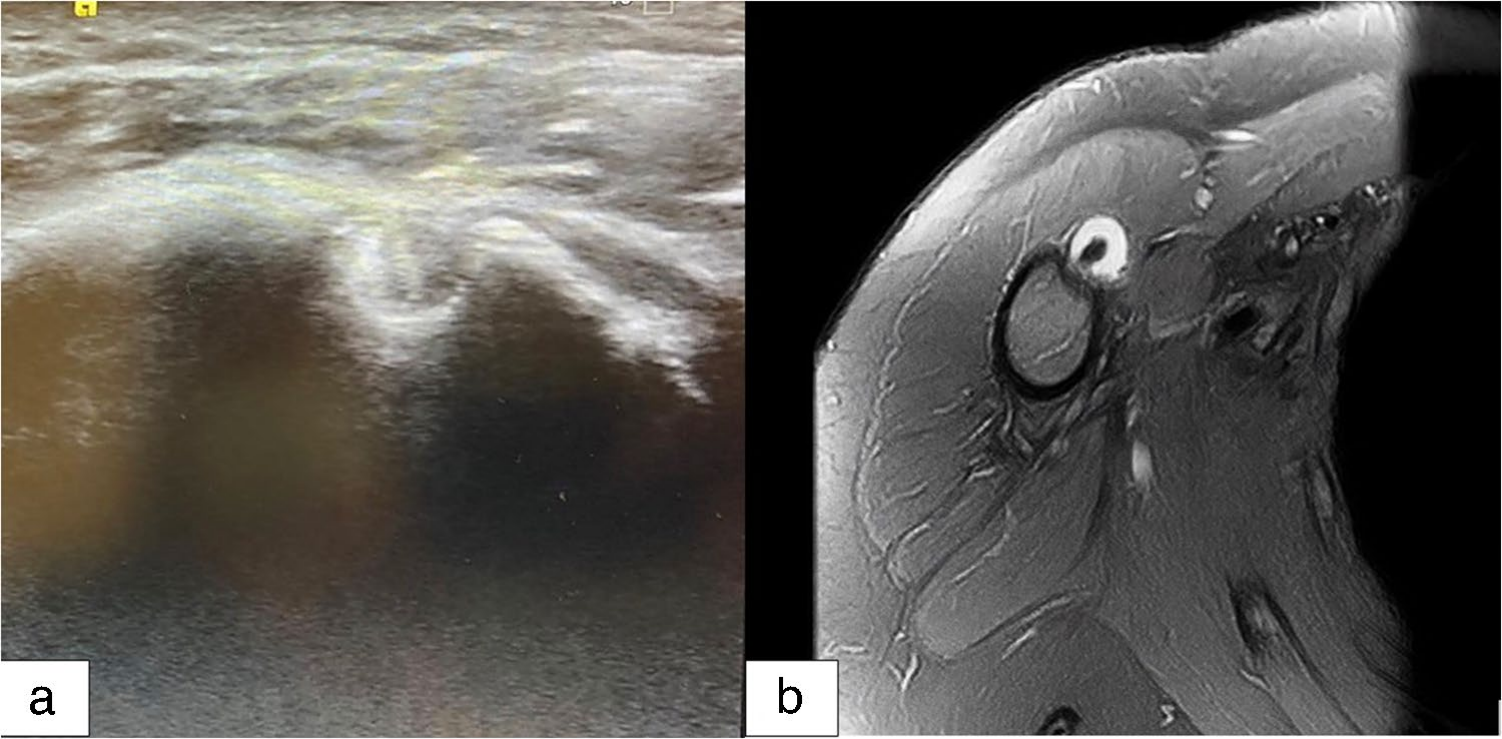
(**a**) Preoperative axial ultrasound image showing long-head biceps tendinopathy. (**b**) Preoperative axial T2-weighted MRI demonstrating increased signal within the LHBT consistent with tendinopathy

Pre-procedural ultrasonographic evaluation was performed using a high-resolution 7–12 MHz linear transducer. The long head of the biceps tendon (LHBT) was examined in both longitudinal and transverse planes to assess overall tendon morphology. Tendon echotexture, sheath effusion, and any signs of delamination were carefully evaluated. The bicipital groove was inspected to determine groove depth and cortical integrity, and dynamic screening was performed to rule out associated subscapularis or supraspinatus pathology that could influence tendon stability or clinical decision-making (Fig. 1).

## Ultrasound-Guided Percutaneous Tenotomy Technique

### Patient Positioning

Patients were positioned supine with the affected arm in 20–30° abduction and slight external rotation, supported comfortably on a side table. This position optimized exposure of the bicipital groove and minimized tension on the tendon.

### Aseptic Preparation

The skin was prepared with 10% povidone–iodine and sterile drapes were applied. A sterile probe cover and sterile ultrasound gel were used throughout the procedure.

### Ultrasound Localization

The LHBT was identified in both transverse and longitudinal planes. Probe adjustments ensured optimal visualization of tendon borders, groove floor, and surrounding soft tissues (Fig. 1).

### Local Anaesthesia

Under in-plane ultrasound guidance, 2–3 mL of 1% lidocaine was injected into the peritendinous sheath. Additional superficial anaesthesia was administered as required. Intratendinous injection was avoided.

### Percutaneous Tenotomy Procedure

All procedures were performed using a standardized ultrasound-guided protocol to ensure reproducibility. A high-resolution linear transducer was positioned in long-axis view to visualize the LHBT continuously. A No. 11 scalpel blade was advanced in-plane, maintaining the cutting edge parallel to the tendon fibres and the probe.

**Fig. 2:**
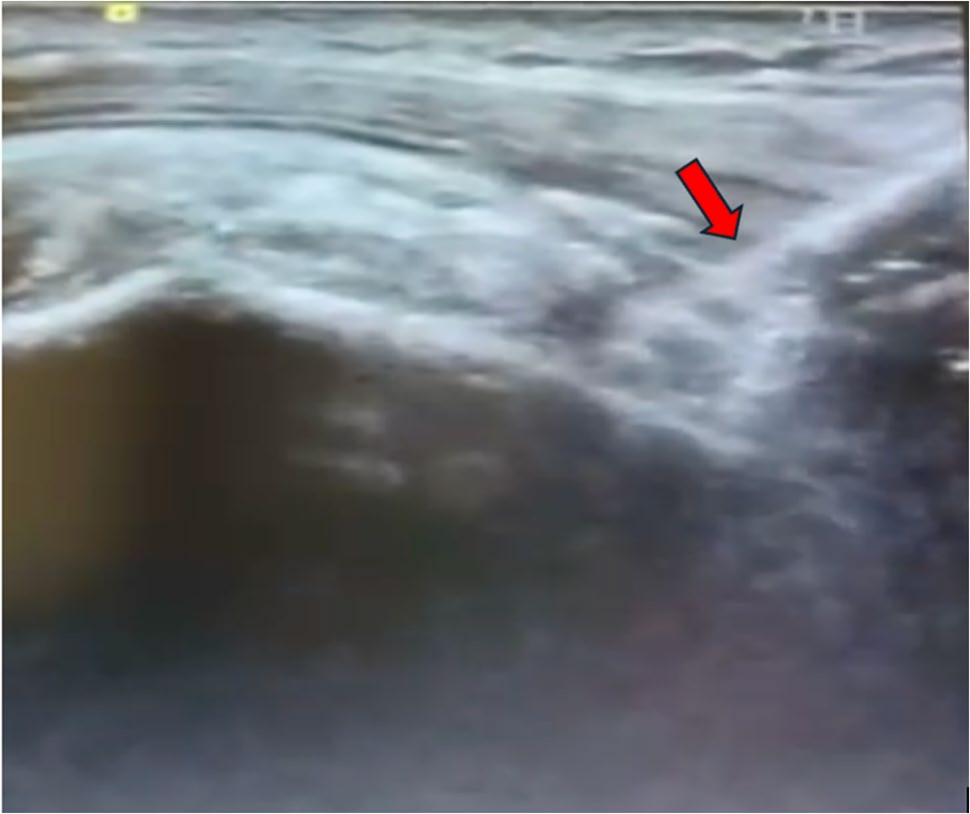
Longitudinal ultrasound image demonstrating percutaneous tenotomy of the long head of the biceps tendon (LHBT) using an 11-blade scalpel. The red arrow indicates the tip of the No. 11 scalpel entering the tendon during the tenotomy procedure

Tenotomy was executed with controlled longitudinal sweeping motions, typically requiring six to ten passes until complete fibre discontinuity was observed. The tendon was released at the proximal bicipital groove, approximately 1–1.5 cm distal to the intra-articular segment, to avoid injury to the rotator interval and subscapularis (Fig. 2).

Throughout the procedure, the entire blade tip was kept in view to minimize risk to the surrounding neurovascular structures. Complete tenotomy was confirmed by (1) loss of fibre continuity, (2) dynamic distal tendon migration during passive pronation–supination, and (3) absence of proximal tension on real-time imaging.

### Completion and Dressing

**Fig. 3:**
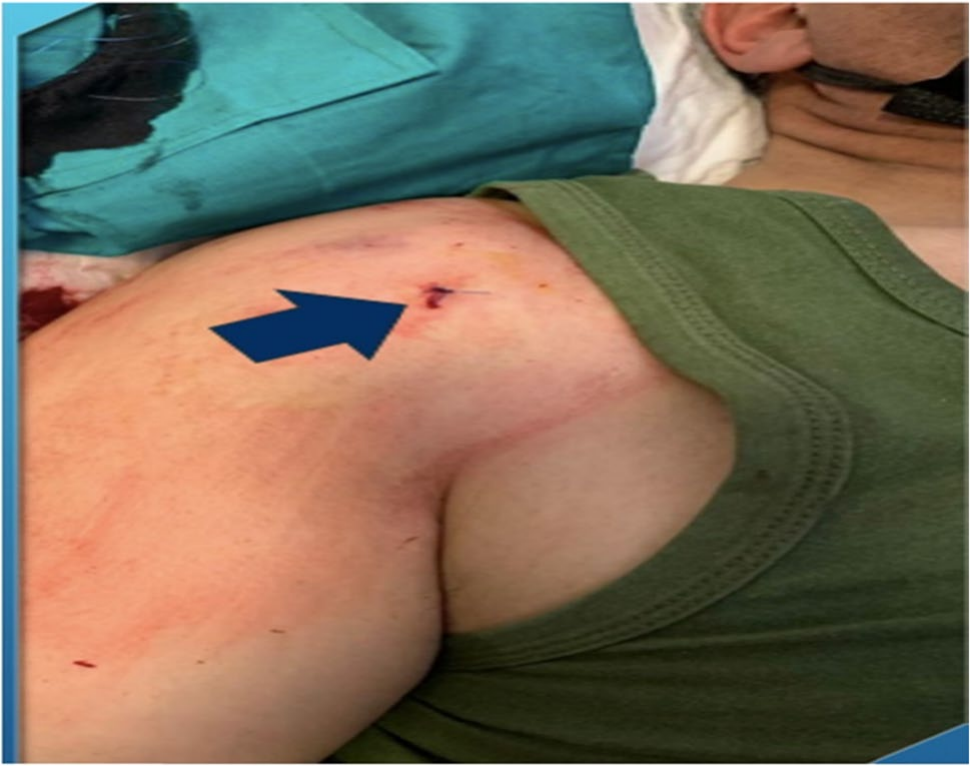
Post-procedural clinical photograph demonstrating the percutaneous tenotomy entry site, closed with a single simple suture (arrow)

Minor bleeding was controlled with brief compression, after which a single 3–0 polypropylene skin suture was placed at the entry site (Fig. 3). A sterile dressing was applied, and no immobilization was required. Patients were observed for 15–20 min following the procedure and discharged the same day.

### Post-Procedural Analgesia

All patients received a standardized analgesia regimen following the procedure. To avoid potential negative effects of non-steroidal anti-inflammatory drugs (NSAIDs) on tendon healing, NSAIDs were not routinely prescribed. Instead, acetaminophen (paracetamol) 500–1000 mg, taken up to three times daily as needed, was recommended as first-line pain control. Short-term NSAID use was permitted only for patients with inadequate response to acetaminophen or contraindications to other analgesics. No patient required opioid medication.

### Post-Procedure Rehabilitation

- 0–48 h: Gentle active range-of-motion exercises
- Week 1–2: Progressive active ROM
- Week 2–6: Strengthening program.
- After 6 weeks: Unrestricted return to activities

No sling immobilization was recommended.

### Outcome Measures and Follow-Up Schedule

All clinical outcomes were assessed at standardized postoperative intervals, including baseline (pre-procedure) and at three, six, 12, and 24 months, with the 24-month visit serving as the final evaluation point. The primary outcome measure was pain intensity, assessed using the Visual Analog Scale (VAS, 0–10 cm). Secondary outcomes included functional assessment with the American Shoulder and Elbow Surgeons (ASES) score and the Constant–Murley score, both of which were recorded at each follow-up time point.

### Tertiary Outcome: Sleep Quality (PSQI)

Sleep quality was evaluated using the Pittsburgh Sleep Quality Index (PSQI), a validated 19-item instrument widely used in musculoskeletal and shoulder research [10]. The PSQI provides a global score ranging from 0 to 21, with higher scores indicating poorer sleep quality; scores greater than 5 were considered reflective of clinically significant sleep disturbance. PSQI assessments were performed at baseline and at three, six, 12-, and 24-month follow-up visits, and only the global PSQI score was used for statistical analysis.

### Complications

All procedure-related complications were recorded, including Popeye deformity, infection, haematoma, cramping, and neurovascular injury.

### Statistical Analysis

Statistical analyses were performed using SPSS version 26 (IBM Corp., Armonk, NY). Data normality was assessed using the Shapiro–Wilk test. Continuous variables were reported as mean ± standard deviation.

Given the repeated measurements of VAS, ASES, Constant–Murley, and PSQI scores across multiple time points, changes over time were analyzed using a repeated-measures ANOVA for normally distributed variables and a Friedman test for non-normal variables. When significant, Bonferroni-adjusted post hoc pairwise comparisons were applied to control for Type I error.

Effect sizes were reported using partial eta-squared (η^2^) for ANOVA and Kendall’s W for non-parametric analyses. A p-value < 0.05 was considered statistically significant. Missing data were handled using pairwise deletion.

A post hoc power analysis using G*Power (version 3.1) confirmed that the sample size provided > 0.95 power to detect the minimal clinically important difference for VAS scores.

## Results

### Patient Characteristics

Fifty-one patients (28 females and 23 males) met the inclusion criteria and completed the full 24-month follow-up. The mean age was 61.8 ± 4.8 years and the mean BMI was 28.7 ± 3.4 kg/m^2^. All patients presented with chronic anterior shoulder pain attributable to LHBT tendinopathy, confirmed on diagnostic ultrasonography. Baseline demographic and clinical characteristics are summarized in Table 1. There were no significant sex-related differences in any baseline clinical scores (all p > 0.05).

**Table 1:** Baseline Demographic and Clinical Characteristics of the Patients

Characteristic	Value
Number of patients, n	51
Female	28 (54.9%)
Male	23 (45.1%)
Mean age, years (mean ± SD)	61.8 ± 4.8
Body mass index, kg/m^2^ (mean ± SD)	28.7 ± 3.4
VAS pain score (baseline), mean ± SD	6.84 ± 1.29
ASES score (baseline), mean ± SD	35.74 ± 10.18
Constant–Murley score (baseline), mean ± SD	60.42 ± 6.23
PSQI global score (baseline), mean ± SD	9.2 ± 3.1

### Pain Outcomes

There was a rapid and substantial reduction in pain following ultrasound guided LHBT tenotomy. Mean VAS scores decreased significantly from baseline to the three month assessment (p < 0.001) and remained consistently low through the six, 12-, and 24-month evaluations (all p < 0.001 vs baseline; Table 2). More than 90% of patients achieved a clinically meaningful improvement of at least two points on the VAS, and pain reduction plateaued between three and six months, demonstrating durable long-term benefit.

**Table 2:** Comparative outcomes of pain, function, and sleep quality at baseline and follow-up intervals

Time point	VAS (mean ± SD)	ASES (mean ± SD)	Constant–Murley (mean ± SD)	PSQI (mean ± SD)	p vs. baseline	Effect Size (η^2^)	MCID Achievement (%)
Baseline	6.84 ± 1.29	35.74 ± 10.18	60.42 ± 6.23	9.2 ± 3.1	—	—	—
3 months	2.06 ± 0.96	80.35 ± 8.78	81.58 ± 2.47	5.1 ± 2.4	< 0.001	**0.71**	**VAS 92%, ASES 88%, Constant 85%, PSQI 76%**
6 months	1.94 ± 0.88	84.26 ± 5.03	82.19 ± 2.85	4.7 ± 2.3	< 0.001	—	—
12 months	2.00 ± 0.78	84.90 ± 5.42	82.29 ± 2.02	4.6 ± 2.1	< 0.001	—	—
24 months	2.16 ± 0.89	85.06 ± 5.02	82.49 ± 1.74	4.8 ± 2.2	< 0.001	—	—

** Statistical analysis was performed using repeated-measures ANOVA (or the Friedman test for non-normal distributions) with Bonferroni-adjusted post hoc comparisons. Effect sizes are presented as partial eta-squared (η^2^). A p-value < 0.05 was considered statistically significant

### Effect Size and MCID Achievement

Effect size analysis demonstrated large improvements across all primary and secondary outcomes. Repeated-measures ANOVA revealed a large effect size for VAS (η^2^ = 0.71), ASES (η^2^ = 0.68), and Constant–Murley scores (η^2^ = 0.64), while PSQI changes showed a moderate-to-large effect (η^2^ = 0.56). Clinically meaningful improvement (meeting or exceeding established MCID thresholds) was achieved in 92.1% of patients for VAS (≥ 2-point reduction), 88.2% for ASES (≥ 12-point increase), 85.3% for Constant–Murley (≥ 10-point increase), and 76.4% for PSQI (≥ 3-point reduction).

### Functional Outcomes

ASES and Constant–Murley scores improved markedly during the early postoperative period. Most functional recovery occurred within the first three months, after which scores stabilized and were maintained through the final follow-up at 24 months. Both ASES and Constant–Murley improvements were statistically significant at all postoperative assessments compared with baseline (p < 0.001 for all; Table 2), indicating sustained restoration of shoulder function.

### Sleep Quality Outcomes

Sleep quality, as measured by the PSQI, significantly improved following the procedure and remained stable during long-term follow-up. PSQI scores demonstrated continuous improvement from baseline to the three month mark, with progressive reductions through six and 12 months and sustained benefit at 24 months (all p < 0.001). The proportion of patients with clinically poor sleep quality (PSQI > 5) decreased from 78.4% at baseline to 29.4% at the 24-month evaluation. Improvements in PSQI were moderately correlated with reductions in VAS pain scores (r ≈ 0.58), suggesting that pain relief contributed meaningfully to enhanced sleep quality (Table 2).

### Complications

Ultrasound-guided percutaneous LHBT tenotomy was not associated with any major complications. Popeye deformity occurred in four patients (7.8%), all of whom remained asymptomatic and required no further intervention. No infections, haematomas, or neurovascular injuries were observed, and no patient required conversion to arthroscopic surgery. Overall, 92.2% of patients experienced no procedure-related complications.

### Patient Satisfaction

At final follow-up, 88.2% of patients reported being satisfied or highly satisfied with the procedure. Satisfaction demonstrated strong correlation with both pain reduction (r ≈ 0.61) and ASES score improvement (r ≈ 0.55), indicating alignment between objective functional gains and subjective patient perception.

## Discussion

The long head of the biceps tendon (LHBT) is a well-recognized source of anterior shoulder pain and frequently coexists with rotator cuff pathology [7–9]. Management of symptomatic LHBT disorders remains controversial, particularly in patients who fail conservative treatment [4]. Arthroscopic tenotomy and tenodesis are established options but require operating room resources and anesthesia, which may be suboptimal for elderly or comorbid patients [11–13]. In this context, ultrasound-guided biceps tenotomy (USG-BT) has emerged as a minimally invasive alternative performed under local anesthesia, offering a simplified outpatient approach [14].

Isolated LHBT pathology is relatively uncommon, particularly in elderly patients, in whom disorders of the long head of the biceps tendon most frequently coexist with rotator cuff disease, pulley lesions, or other intra-articular pathology [15]. Recent International Orthopaedics research has further demonstrated that age-related patterns of rotator cuff tears are frequently accompanied by progressive pulley and adjacent structural lesions, underscoring the increasing complexity of shoulder pathology with advancing age [16]. Previous studies have demonstrated the diagnostic difficulty of LHBT pathology, even in the presence of rotator cuff tears, with limited concordance between imaging findings and intra-operative assessment, underscoring the complexity of accurately identifying truly isolated cases [17]. Consequently, isolated LHBT tendinopathy represents a clinical challenge and carries a substantial risk of misclassification in the absence of comprehensive clinical and imaging evaluation [18].

For this reason, strict patient selection constituted a central methodological focus of the present study. A multimodal diagnostic approach combining targeted clinical examination, high-resolution ultrasonography, and standardized MRI was used to confirm isolated LHBT involvement and to exclude concomitant rotator cuff or instability pathology. While this strategy strengthens the internal validity of our findings, it also limits generalizability to a highly selected population. Nevertheless, given the rarity of truly isolated LHBT tendinopathy, meticulous diagnostic work-up and careful patient selection should be considered essential when evaluating candidates for ultrasound-guided percutaneous tenotomy [19].

In the present study, USG-BT resulted in significant and sustained improvements in pain and shoulder function. VAS scores decreased rapidly and remained low throughout follow-up, while ASES and Constant scores improved markedly and stabilized after the early postoperative period. These findings are consistent with previous reports demonstrating favourable clinical outcomes following ultrasound-guided percutaneous tenotomy in chronic tendinopathies [20, 21].

A key advantage of ultrasound guidance is the ability to perform real-time visualization of the tendon and surrounding structures, allowing accurate targeting and confirmation of complete release while minimizing the risk of iatrogenic injury [22, 23]. The minimally invasive nature of the technique and the use of local anaesthesia likely contribute to the rapid functional recovery observed in our cohort.

Popeye deformity occurred in 7.8% of patients, consistent with rates reported after arthroscopic tenotomy [9, 24], and was asymptomatic in all cases. No major complications were observed, supporting the safety profile of the ultrasound-guided approach. Compared with tenodesis, which carries implant-related risks and typically requires longer rehabilitation, tenotomy offers technical simplicity and faster recovery in appropriately selected patients [25].

Sleep disturbance is an often underappreciated aspect of LHBT pathology. In our study, PSQI scores improved significantly, with a substantial reduction in the proportion of patients reporting clinically poor sleep. The moderate correlation between pain reduction and sleep improvement highlights the relevance of night pain relief in this patient population and is consistent with previous findings in shoulder disorders [26].

Successful execution of USG-BT requires familiarity with musculoskeletal ultrasound and shoulder anatomy. Maintaining continuous visualization of the blade tip and using a strict in-plane approach are essential to ensure procedural safety and reproducibility [27].

This study has several limitations. Its retrospective design and lack of a control group limit direct comparison with arthroscopic techniques. The sample size was modest, and postoperative imaging was not routinely performed to confirm tendon retraction. Additionally, the strict selection of patients with isolated LHBT tendinopathy, while strengthening diagnostic accuracy, may limit generalizability. Larger prospective comparative studies are needed to further define the long-term role of this technique. Furthermore, recent publications have emphasized the importance of cautious interpretation of outcomes and methodological awareness in musculoskeletal research, particularly in non-comparative and retrospective studies [28].

Despite these limitations, the consistent improvements in pain, function, and sleep quality, together with the low complication rate, suggest that USG-BT may represent a valuable minimally invasive option for carefully selected patients with isolated LHBT tendinopathy.

## Conclusion

Ultrasound-guided percutaneous tenotomy provides safe, rapid, and durable improvement in pain, function, and sleep quality for patients with isolated LHBT tendinopathy who fail conservative treatment. The procedure’s minimally invasive nature, outpatient feasibility, and low complication rate make it a valuable alternative to arthroscopic techniques. Larger prospective comparative studies are warranted to further define its long-term role in shoulder tendon pathology.

## Data Availability

“The clinical datasets supporting the findings of this study are derived from patient medical records and contain protected health information. For this reason, they cannot be shared publicly. De-identified data may be made available by the corresponding author upon reasonable request and with institutional approval.”
